# Supplementary material for: Effectiveness of Remote Interventions to Improve Medication Adherence in Patients after Stroke: A Systematic Literature Review and Meta-Analysis
Source: Behav Sci (Basel). 2023 Mar 11;13(3):246. doi: 10.3390/bs13030246 (PMC10044982; doi:10.3390/bs13030246)
Supplement: Supplementary file 1 [file behavsci-13-00246-s001.zip › behavsci-2249849-supplementary.pdf]

Supplementary Material

Table S1. Search Strategy

#1 (stroke OR ischaemic stroke OR haemorrhagic stroke OR transient ischaemic stroke OR poststroke OR cerebrovascular disorders OR basal ganglia cerebrovascular disease OR brain ischemia OR carotid artery diseases OR cerebral small vessel diseases OR intracranial arterial diseases or brain infarction OR subarachnoid haemorrhage).ab

AND

#2 (mHealth OR SMS text OR mobile applications OR cell phone OR social media OR smartphone OR apps OR automated phone calls OR telemedicine OR remote consultations OR medical technology).ab

AND

#3 medication adherence OR medication compliance OR patients adherence OR patients compliance OR persistence OR medic*

AND

#4 control trial OR randomized controlled trial or random*

Table S2. Baseline characteristics of included studies

Author, Setting	Population characteristics (n, Average Age, % of Male, Eligibility criteria)	Duration of Study	Delivery Mode	Outcome Measures
***Adie et al*., 2010**<br>**UK**	N = 56, 72.5 y, 46% male<br>Diagnosis of minor stroke or TIA within one month,<br>clinic SBP ≥ 140 mmHg	6 months	Telephone	Behavioural: Medication adherence<br>Clinical: sBP, dBP, LDL-C
***Hedegaard et al*., 2014**<br>**Denmark**	N = 211, 67 y, 61% male<br>Diagnosis with acute first-time ischaemic stroke or TIA within the previous 30 days	12 months	Telephone	Behavioural: Medication adherence
***Kamal et al*., 2015**<br>**Pakistan**	N = 200, 56.8 y, 67.5% male<br>Diagnosis of stroke, use at least two drugs to control risk factors of stroke	2 months	Automated SMS text messages	Behavioural: Medication adherence<br>Clinical: dBP

***Kamal et al*., 2018**<br>**Pakistan**	N = 201, 58.4 y, 77.2% male<br>Diagnosis of cardiovascular disease or cerebrovascular accident	3 months	Videos, mobile application	Behavioural: Medication adherence
***Kamal et al*., 2020**<br>**Pakistan**	N = 310, 60.2 y, 67.4% male<br>Diagnosis of first-time acute stroke with designated caregiver	12 months	Telephone and SMS text messages	Clinical: dBP, sBP, LDL-C
***Labovitz et al*., 2017**<br>**USA**	N = 28, 57 y, 46.4% male<br>Diagnosis of ischaemic stroke, receiving anticoagulation drugs	12 weeks	Mobile application	Behavioural: Medication adherence
***Lakshminarayan et al*., 2018**<br>**USA**	N = 56, 65.6 y, 72% male<br>Diagnosis of acute ischaemic stroke or intra-parenchymal haemorrhage	90 days	Telephone and email	Behavioural: Medication adherence<br>Clinical: sBP
***Ogren et al*., 2018**<br>**Sweden**	N = 871, 69.6 y, 59.2% male<br>Diagnosis of ischaemic stroke, TIA or intracerebral hematoma	36 months	Telephone	Clinical: sBP, dBP, LDL-C

***Wan et al*., 2016**<br>**China**	N = 91, 59.7 y, 71.3% male<br>Diagnosis of ischaemic stroke	6 months	Telephone	Behavioural: Medication adherence
***Yan et al*., 2021**<br>**China**	N = 1299, 65.7 y, 57.4%<br>Diagnosis of stroke	12 months	Voice message and automated SMS text messages	Behavioural: Medication adherence<br>Clinical: sBP, dBP

Abbreviations: Systolic blood pressure (sBP), Diastolic blood pressure (dBP), Transient ischaemic stroke (TIA), Low-density lipoprotein (LDL-C)

Table S3. Behavioural analysis of the intervention components

Study	Intervention group	Comparator group
***Adie et al*., 2010**	**Description:** Participants received telephone-based counselling that targeted their specific goals regarding lifestyle changes. These discussions reviewed BP, cholesterol control, medication, smoking, diet and physical activity. Educational materials tailored to the patients' needs were also provided.<br><br>**Delivery mode:** Telephone<br>**Length and frequency:** 20-minute call at 7-10 days, 1, 2 and 4 months<br>**Tailored advice:** Yes<br>**COM-B:** *Motivation* – setting goals and outcome expectations; *Opportunity* – provided social support	**Description:** Usual care with GP<br><br>**Delivery mode:** Face-to-face<br>**Length and frequency:** 6 months
***Hedegaard et al*., 2014**	**Description:** Participants received focused medication review and an interview, then were followed up with telephone calls after discharge<br><br>**Delivery mode:** Telephone<br>**Length and frequency:** Contacted 1 week, 2 and 6 months after discharge<br>**Tailored advice:** Yes<br>**COM-B:** *Motivation* – Telephone follow-up on patients' plans that were set during motivational interviews, addressed any new medication problems, checked their medication adherence and summarised their progress according to their agreed plans; *Opportunity* – provided social support	**Description:** Standard care with short counselling about patients' lifestyles<br><br>**Delivery mode:** Face-to-face<br>**Length and frequency:** 12 months
***Kamal et al*., 2015**	**Description**: Participants received automated SMS text message reminders according to their	**Description**: Usual care provided at the centre for stroke patients, including regular

	prescriptions. The participants were then required to reply to the text message regarding if they had taken their medication or not. SMS messages containing health information were also sent weekly to the participants, which were tailored according to the patients' drug and medical profiles.<br><br>**Delivery mode:** SMS text messages<br>**Length and frequency:** Reminder texts according to individuals' prescriptions and health information SMS sent weekly for 12 months<br>**Tailored advice:** Yes<br>**COM-B:** *Capability* – Reminders to improve cognitive functions	follow-up visits with stroke neurologists. Patients were provided with the telephone number of the stroke team in case of emergency, and they also received SMS messages or phone calls reminding them of their clinic appointments 1-2 days before<br><br>**Delivery mode**: Face-to-face, SMS/phone call<br>**Length and frequency**: 12 months
***Kamal et al*., 2018**	**Description**: Participants received daily interactive voice response call services, tailored medication reminders, and lifestyle modification messages per week<br><br>**Delivery mode:** Telephone and SMS text message<br>**Length and frequency:** Daily calls, and weekly SMS messages for 3 months<br>**Tailored advice**: Yes<br>**COM-B**: *Capability* – improving patients' comprehension of lifestyle changes to prevent recurrent stroke, and improving cognitive functions by reminding medication adherence through SMS text messages	**Description**: Usual care and regular follow-up with stroke neurologist or cardiologist, also provided helpline number in case of emergency or queries about illness or medications<br><br>**Delivery mode**: Face-to-face<br>**Length and frequency**: 3 months
***Kamal et al*., 2020**	**Description**: Participants were sent reminder messages to watch movies at home. Videos were shown to patients at time of discharge on	**Description**: Usual care, patients given information booklets and verbal instructions before discharge. Follow-up

	emergency preparedness, followed by second and third videos delivered after discharge covering frequently used medications, and strategies to prevent recurrent stroke<br><br>**Delivery mode**: SMS text message, mobile application<br>**Length and frequency**: Reminder messages sent twice a week, and videos delivered first and third month of follow-up after discharge<br>**Tailored advice**: No<br>**COM-B**: *Capability* – improve patients' comprehension of disease and prescribed treatment, and improving patients' cognitive functions by sending reminder text messages	appointments were provided after discharge<br><br>**Delivery mode**: Face-to-face<br>**Length and frequency**: 12 months
***Labovitz et al*., 2017**	**Description**: AI platform AiCure monitored participants' ingestions of prescribed anticoagulants. Any incorrect or missed doses were flagged on the AI platform, and the patient would be contacted by a study coordinator or the AiCure team<br><br>**Delivery mode**: Mobile application<br>**Length and frequency**: 12 weeks<br>**Tailored advice**: No<br>**COM-B**: *Capability* – reminder to improve cognitive functions	**Description**: Usual care<br><br>**Delivery mode**: Face-to-face<br>**Length and frequency**: 12 weeks
***Lakshminarayanan et al*., 2018**	**Description**: Participants were provided with a wireless BP monitor and a smartphone to measure their daily BP. The data was automatically transmitted to a database monitored by	**Description**: Participants given BP monitor that did not transmit data automatically, and only shared BP data during clinic visits

	investigators, who then adjusted patients' medication prescription. Investigators communicated with patients via telephone and email to adjust medication prescription and provided advice on lifestyle modifications to improve adherence.<br><br>**Delivery mode**: telephone, email<br>**Length and frequency**: BP measured prior to breakfast, coffee or medication every day, participants' BP monitored biweekly for 90 days<br>**Tailored advice**: Yes<br>**COM-B**: *Opportunity* – provided social support; *Capability* – reminder to improve cognitive functions	**Delivery mode**: Face-to-face<br>**Length and frequency**: 90 days
***Ogren et al*., 2018**	**Description**: Participants received counselling and pharmacological treatment assessments via telephone. Medical treatments were adjusted by physicians if LDL-C and/or BP targets were not reached<br><br>**Delivery mode**: Telephone<br>**Length and frequency**: Physician consulting every 4 weeks when necessary for 36 months<br>**Tailored advice**: Yes<br>**COM-B**: *Motivation* – ensured that patients had correct perceptions of the illness and corrected their beliefs about treatment, encouraging outcome expectancies; *Opportunity* – provided social support	**Description**: In-hospital treatment and secondary preventative care after discharge based on local standards<br><br>**Delivery mode**: Face-to-face<br>**Length and frequency**: 36 months
***Wan et al*., 2016**	**Description**: Participants received telephone follow-up conducted by stroke nurses. The calls	**Description**: Usual care that included educational brochures on stroke and its

	were structured, guideline-based and included goal-setting advice<br><br>**Delivery mode**: Telephone<br>**Length and frequency**: 15 to 20-minute telephone calls at 1 weeks, 1 and 3 months after discharge<br>**Tailored advice**: Yes<br>**COM-B**: *Motivation* – by setting goals and outcome expectations related to lifestyle changes according to each patients' pre-stroke lifestyles; *Opportunity* – provided social support	risks. Routine measurements of BP and medication adherence after discharge<br><br>**Delivery mode**: Face-to-face<br>**Length and frequency**: 6 months
***Yan et al*., 2021**	**Description**: Participants received daily voice messages or text messages that reminded them medication adherence and physical adherence along with tips<br><br>**Delivery mode**: SMS text messages, voice messages<br>**Length and frequency**: Daily messages for 12 months<br>**Tailored advice**: No<br>**COM-B**: *Capability* – reminder to improve cognitive functions	**Description**: Usual care<br><br>**Delivery mode**: Face-to-face<br>**Length and frequency**: 12 months

Table S4. Included outcomes of eligible studies in the meta-analysis.

Continuous outcomes								
			Mean		SD		Number of participants (n)	
**Study**	**Outcome**	**Measure**	**Intervention**	**Comparator**	**Intervention**	**Comparator**	**Intervention**	**Comparator**
Adie et al., 2010	Medication adherence	Medication knowledge (%)	34	-9	60.1	48	29	27
	Systolic BP	mm Hg	-2.6	-4.6	20.3	19	29	27
	Diastolic BP	mm Hg	-10	-8.4	13	13.2	29	27
	Cholesterol	mmol/L	-0.9	-1	1.2	1	29	27
Hedegaard et al., 2014	Medication adherence	Composite medication possession ratio	0.06	0.12	0.7	0.9	100	100
Kamal et al., 2015	Medication adherence	MMAS scores	0.8	0.1	0.16	0.88	83	79
Kamal et al., 2018	Medication adherence	MMAS scores	0.66	0.4	1.15	1.32	50	47
Labovitz et al., 2017	Medication adherence	Cumulative adherence based on pill count	97.2	90.6	4.4	5.8	15	12
Lakshminarayan et al., 2018	Systolic BP	mm Hg	-9.8	-5.9	14.47	16.68	28	22
	Medication adherence	Mean knowledge scores	2.3	2.6	0.8	0.8	27	19
Ogren et al., 2018	Systolic BP	mm Hg	-8.8	-3	17.6	19.2	320	340

	Diastolic BP	mm Hg	-5.5	-1.4	13.2	12.7	320	340
	LDL-C	mmol/L	-0.3	0.1	1.1	1.1	320	340
Wan et al., 2016	Medication adherence	HPLP II scores	0.67	0.17	0.77	0.73	40	40
Yan et al., 2021	Systolic BP	mm Hg	-7.1	-4.3	18.5	18.9	611	615
	Diastolic BP	mm Hg	-3.9	-2.3	9.6	9.6	611	615
				**Dichotomous outcomes**				
			**Events**			**Total**		
**Study**	**Outcome**	**Measure**	**Intervention**	**Comparator**		**Intervention**	**Comparator**	
Hedegaard et al., 2014	Medication adherence	No. of adherent patients in year of follow-up	74	80		102	101	
Kamal et al., 2020	Systolic BP (goal)	No. of patients	18	11		54	52	
	Diastolic BP (goal)	No. of patients	44	37		54	52	
	LDL-C (goal)	No. of patients	36	30		51	51	
Labovitz et al., 2017	Medication adherence	No. of patients above goal drug concentration level	10	3		10	9	
Lakshminarayanan et al., 2018	Systolic BP (goal)	No. of patients	23	14		28	22	

Ogren et al., 2018	Systolic BP (goal)	No. of patients	254	188		320	340
	Diastolic BP (goal)	No. of patients	289	265		320	340
	LDL-C (goal)	No. of patients	222	166		320	340
Yan et al., 2021	Medication adherence	MGLS score	824	671		1134	1018

Table S5. Coding of intervention acceptability

Study	Measurement	Results
Lakshminarayan et al., 2018	Estimated acceptability by measuring elements of intervention usability of the interventions using the Marshfield Usability Survey (65) that is based on the Likert scale (1 = Strongly disagree, 5 = Strongly agree). They also asked patients about technical difficulties they faced while using the intervention as well as for unstructured feedback and suggestions for changes at the end of the trial.	High patient satisfaction with the interventions was found as most participants found it easy to use and convenient and thus, did not require additional help to use the system. They also reported that overall unstructured feedback was positive as participants liked the "instantaneous automatic transmission" of data directly to their healthcare providers. Some negative comments stated issues with technology such as phone running out of battery or confusion with the applications. Recommendations from patients included need for more training and clearer instructions for using the intervention.
Labovitz et al., 2017	Asked patients in the intervention group to complete a four-question satisfaction and usability questionnaires pre- and post-study.	Reported a 10% increase from 73.3% to 83.3% of patient satisfaction.
Kamal et al., 2015	Asked patients to complete two questionnaires, including a self-reported questionnaire and another that reported satisfaction based on previous literature (66).	Recorded 96.07% patient satisfaction and 95.6% acceptability of the mHealth intervention.
Kamal et al., 2018	Conducted open-ended interviews asking participants about their opinions and recommendations on	Found that majority of patients were satisfied with the intervention as they found it "excellent", "useful", "convenient" and "informative".

	how to further improve the interventions.	Patients suggested to reduce total duration of calls, simplify information provided to them, add pictorial messages and to add local languages to increase accessibility of the intervention in local communities.
Hedegaard et al., 2014	Asked patients to rate their satisfaction with specific intervention elements, including their views on the received medication adherence care.	Majority of patients were "very satisfied" with the intervention.

Study or Subgroup	Intervention Mean	Intervention SD	Intervention Total	Comparator Mean	Comparator SD	Comparator Total	Weight	Std. Mean Difference IV, Random, 95% CI
**1.1.3 Capability**								
Kamal, et al. 2015	0.8	0.16	83	0.1	0.88	79	10.2%	1.11 [0.78, 1.45]
Kamal, et al. 2018	0.66	1.15	50	0.4	1.32	47	9.7%	0.21 [−0.19, 0.61]
Labovitz, et al. 2017	97.2	4.4	15	90.6	5.8	12	6.5%	1.26 [0.42, 2.11]
Lakshminarayan, et al. 2018	2.3	0.8	27	2.6	0.8	19	8.3%	−0.37 [−0.96, 0.22]
Subtotal (95% CI)			175			157	34.7%	0.54 [−0.18, 1.26]
Heterogeneity: $Tau^2 = 0.46$; $Chi^2 = 25.57$, df = 3 ($P < 0.0001$); $I^2 = 88\%$								
Test for overall effect: Z = 1.47 (P = 0.14)								
**1.1.4 Opportunity**								
Adie, et al. 2010	34	60.1	29	−9	48	27	8.6%	0.78 [0.23, 1.32]
Hedegaard, et al. 2014	0.06	0.7	100	0.12	0.9	100	10.5%	−0.07 [−0.35, 0.20]
Lakshminarayan, et al. 2018	2.3	0.8	27	2.6	0.8	19	8.3%	−0.37 [−0.96, 0.22]
Wan, et al. 2016	0.67	0.77	40	0.17	0.73	40	9.4%	0.66 [0.21, 1.11]
Subtotal (95% CI)			196			186	36.8%	0.25 [−0.26, 0.75]
Heterogeneity: $Tau^2 = 0.21$; $Chi^2 = 15.44$, df = 3 ($P = 0.001$); $I^2 = 81\%$								
Test for overall effect: Z = 0.95 (P = 0.34)								
**1.1.5 Motivation**								
Adie, et al. 2010	34	60.1	29	−9	48	27	8.6%	0.78 [0.23, 1.32]
Hedegaard, et al. 2014	0.06	0.7	100	0.12	0.9	100	10.5%	−0.07 [−0.35, 0.20]
Wan, et al. 2016	0.67	0.77	40	0.17	0.73	40	9.4%	0.66 [0.21, 1.11]
Subtotal (95% CI)			169			167	28.5%	0.42 [−0.17, 1.01]
Heterogeneity: $Tau^2 = 0.22$; $Chi^2 = 11.98$, df = 2 ($P = 0.003$); $I^2 = 83\%$								
Test for overall effect: Z = 1.41 (P = 0.16)								
**Total (95% CI)**			540			510	100.0%	0.40 [0.07, 0.72]
Heterogeneity: $Tau^2 = 0.24$; $Chi^2 = 62.85$, df = 10 ($P < 0.00001$); $I^2 = 84\%$								
Test for overall effect: Z = 2.38 (P = 0.02)								
Test for subgroup differences: $Chi^2 = 0.47$, df = 2 ($P = 0.79$), $I^2 = 0\%$								

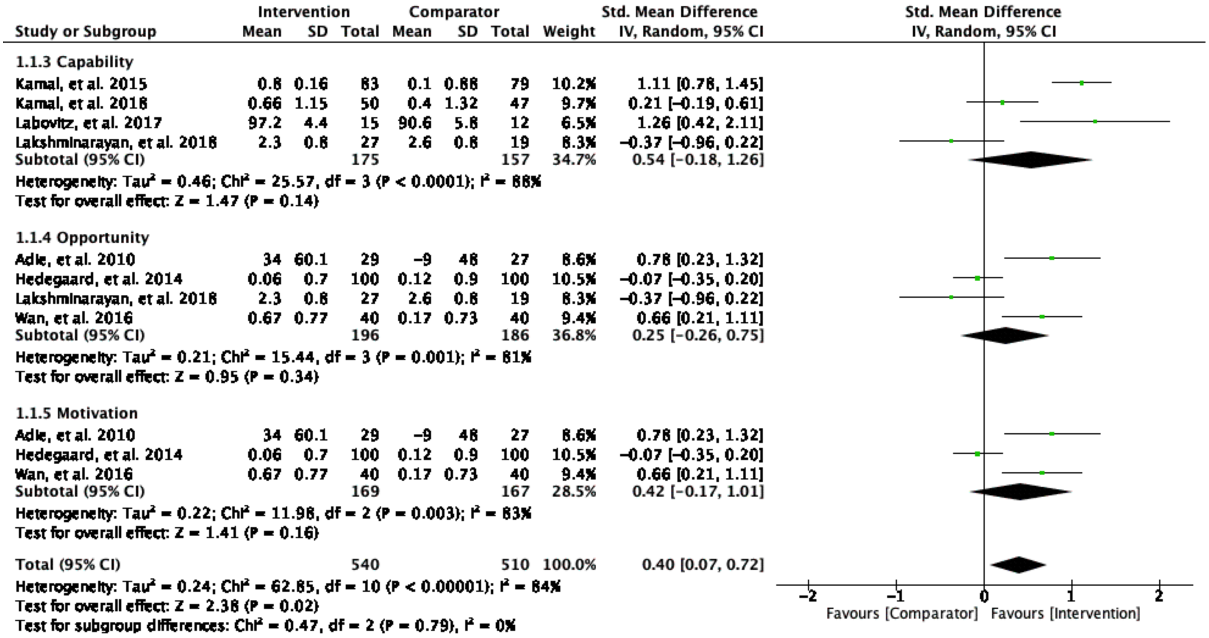

Figure S1. Subgroup analysis, intervention content, continuous outcome

Study or Subgroup	Intervention Events	Intervention Total	Comparator Events	Comparator Total	Weight	Odds Ratio M-H, Random, 95% CI
**2.1.3 Capability**						
Labovitz, et al. 2017	10	10	3	9	3.9%	39.00 [1.72, 883.62]
Yan, et al. 2021	824	1134	671	1018	38.9%	1.37 [1.14, 1.65]
Subtotal (95% CI)		1144		1027	42.8%	5.03 [0.20, 123.32]
Total events	834		674			
Heterogeneity: $Tau^2 = 4.34$; $Chi^2 = 4.42$, df = 1 ($P = 0.04$); $I^2 = 77\%$						
Test for overall effect: Z = 0.99 (P = 0.32)						
**2.1.4 Opportunity**						
Hedegaard, et al. 2014	74	102	80	101	28.6%	0.69 [0.36, 1.33]
Subtotal (95% CI)		102		101	28.6%	0.69 [0.36, 1.33]
Total events	74		80			
Heterogeneity: Not applicable						
Test for overall effect: Z = 1.11 (P = 0.27)						
**2.1.5 Motivation**						
Hedegaard, et al. 2014	74	102	80	101	28.6%	0.69 [0.36, 1.33]
Subtotal (95% CI)		102		101	28.6%	0.69 [0.36, 1.33]
Total events	74		80			
Heterogeneity: Not applicable						
Test for overall effect: Z = 1.11 (P = 0.27)						
**Total (95% CI)**		1348		1229	100.0%	1.06 [0.55, 2.02]
Total events	982		834			
Heterogeneity: $Tau^2 = 0.27$; $Chi^2 = 12.03$, df = 3 ($P = 0.007$); $I^2 = 75\%$						
Test for overall effect: Z = 0.17 (P = 0.86)						
Test for subgroup differences: $Chi^2 = 1.44$, df = 2 ($P = 0.49$), $I^2 = 0\%$						

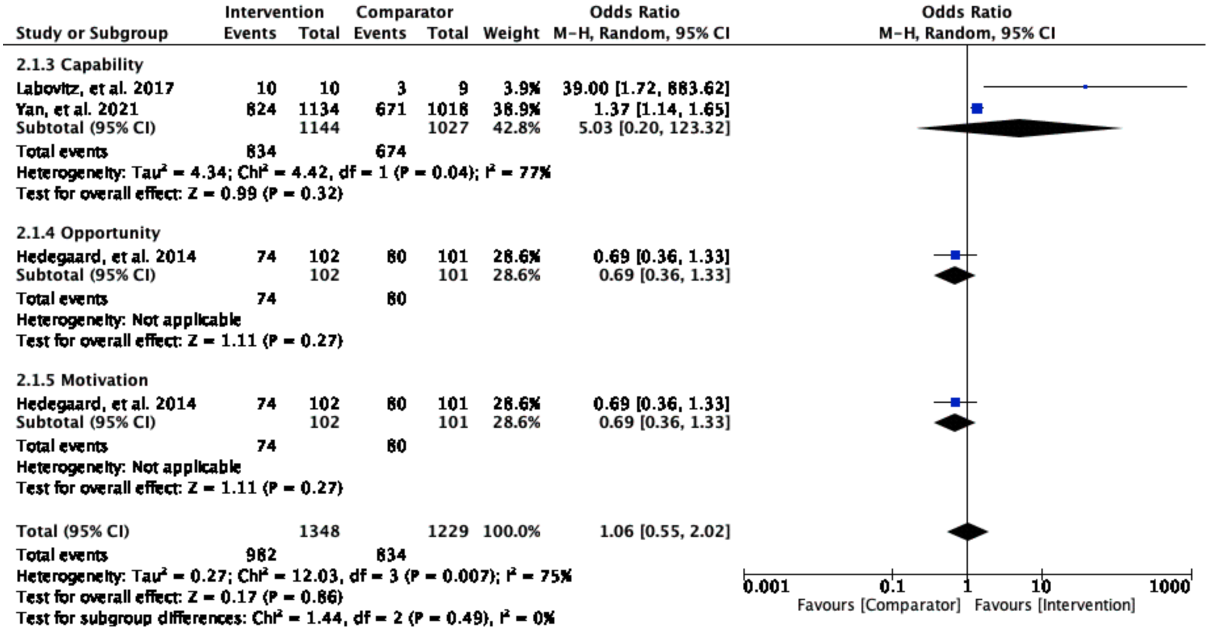

Figure S2. Subgroup analysis, intervention content, dichotomous outcome

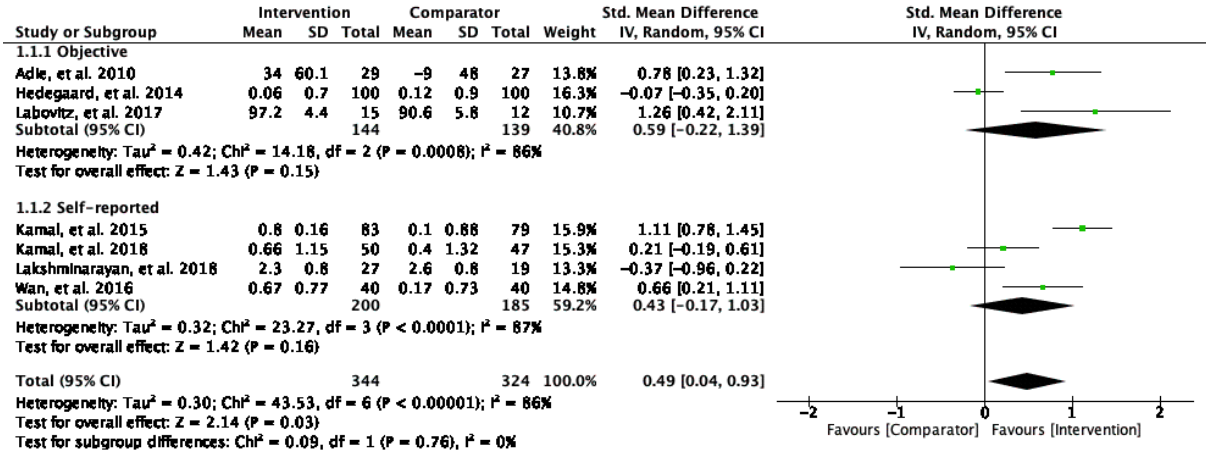

Figure S3. Subgroup analysis, outcome measurement, continuous outcome

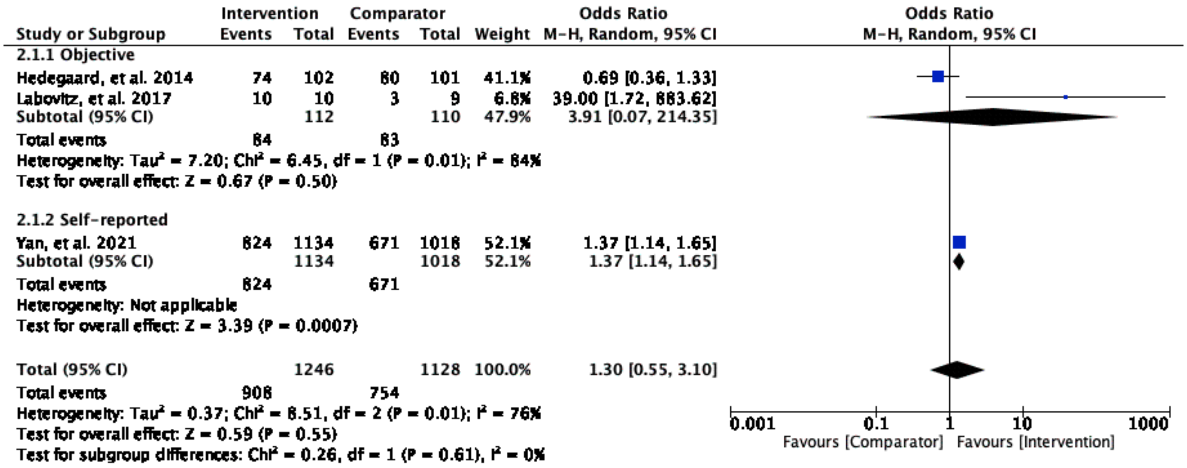

Figure S4. Subgroup analysis, outcome measurement, dichotomous outcome

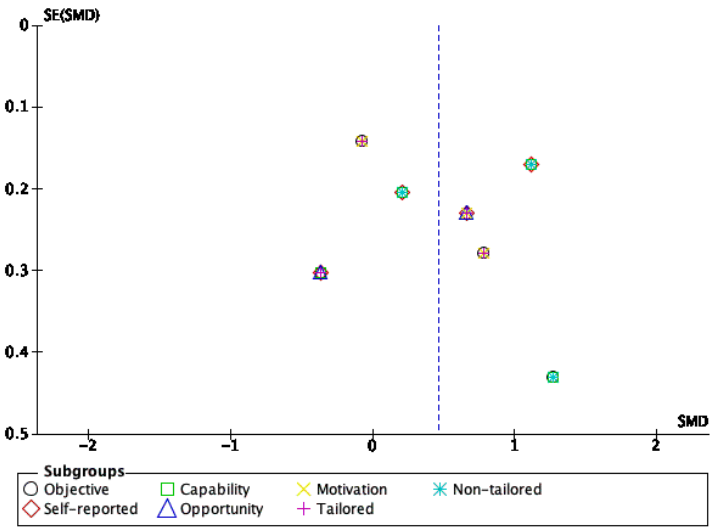

Figure S5. Funnel plot for adherence continuous outcomes

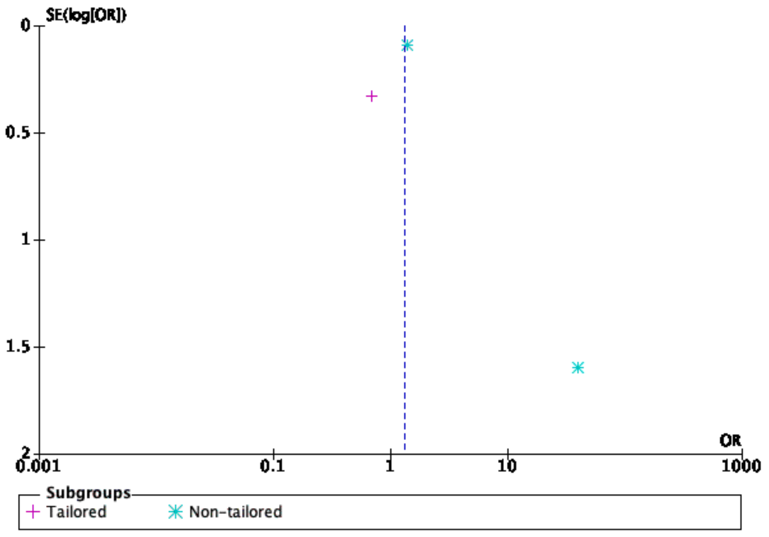

Figure S6. Funnel plot for adherence dichotomous outcomes

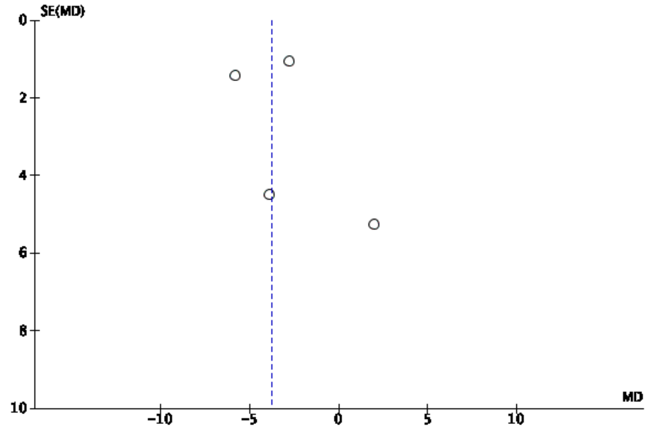

Figure S7. Funnel plot for sBP continuous outcomes

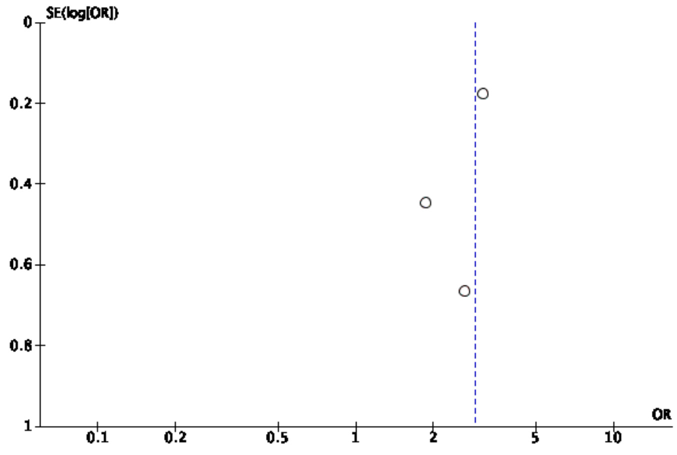

Figure S8. Funnel plot for sBP dichotomous outcomes

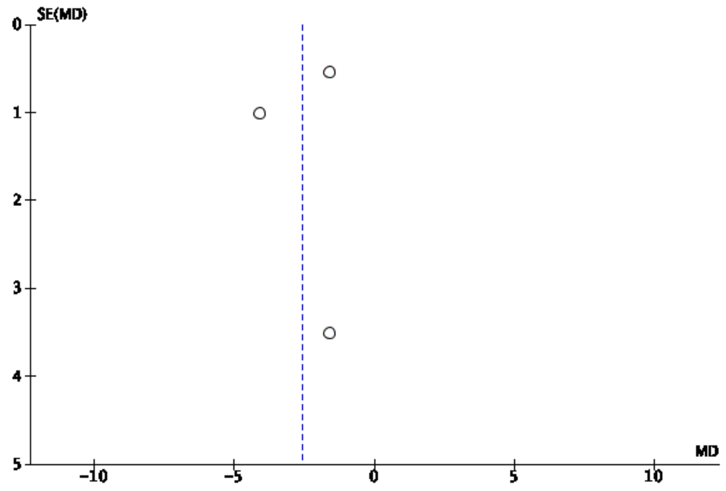

Figure S9. Funnel plot for dBP continuous outcomes

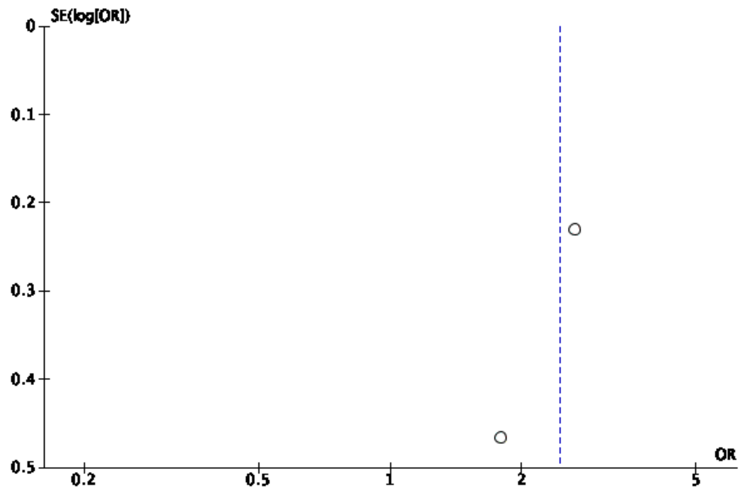

Figure S10. Funnel plot for dBP dichotomous outcomes

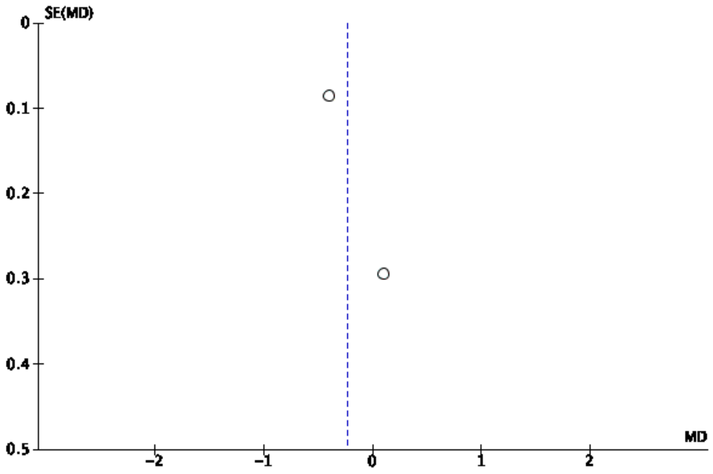

Figure S11. Funnel plot for LDL-C continuous outcomes

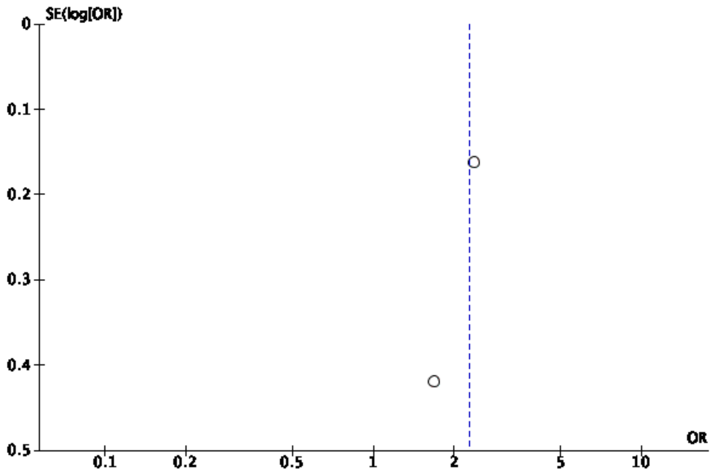

Figure S12. Funnel plot for LDL-C dichotomous outcomes
